# Modifications of sodium channel voltage dependence induce arrhythmia-favouring dynamics of cardiac action potentials

**DOI:** 10.1371/journal.pone.0236949

**Published:** 2020-08-04

**Authors:** Pia Rose, Jan-Hendrik Schleimer, Susanne Schreiber

**Affiliations:** 1 Institute for Theoretical Biology, Humboldt-Universität zu Berlin, Berlin, Germany; 2 Bernstein Center for Computational Neuroscience, Berlin, Germany; University of Minnesota, UNITED STATES

## Abstract

Heart arrhythmia is a pathological condition where the sequence of electrical impulses in the heart deviates from the normal rhythm. It is often associated with specific channelopathies in cardiac tissue, yet how precisely the changes in ionic channels affect the electrical activity of cardiac cells is still an open question. Even though sodium channel mutations that underlie cardiac syndromes like the Long-Q-T and the Brugada-syndrome are known to affect a number of channel parameters simultaneously, previous studies have predominantly focused on the persistent late component of the sodium current as the causal explanation for an increased risk of heart arrhythmias in these cardiac syndromes. A systematic analysis of the impact of other important sodium channel parameters is currently lacking. Here, we investigate the reduced ten-Tusscher-model for single human epicardium ventricle cells and use mathematical bifurcation analysis to predict the dependence of the cardiac action potential on sodium channel activation and inactivation time-constants and voltage dependence. We show that, specifically, shifts of the voltage dependence of activation and inactivation curve can lead to drastic changes in the action potential dynamics, inducing oscillations of the membrane potential as well as bistability. Our results not only demonstrate a new way to induce multiple co-existing states of excitability (biexcitability) but also emphasize the critical role of the voltage dependence of sodium channel activation and inactivation curves for the induction of heart-arrhythmias.

## Introduction

Many channelopathies are known to increase the risk for heart-arrhythmias [1]. Such ion channel mutations hinder the coordinated conduction of electrical signals in the heart by changes they incur for the action potential shape [1,2]. For example, both gain- and loss-of-function mutations in Na_V_1.5 channels (which have been associated with the Long-Q-T and Brugada-syndromes [3]) can be a risk-factor for heart-arrhythmias because of the resulting prolongation or shortening of the cardiac action potential [1]. Even though these sodium channel mutations are likely to impact several of the channel’s electrophysiological parameters simultaneously [4–6], so far the majority of studies have focused on the late sodium current as the most crucial parameter for triggering heart-arrhythmias [7–9]. Along these lines, the overlap between the activation and inactivation curve and its resulting window current are discussed as one possible mechanism to increase the late sodium current [10]. The window current, however, has a different voltage-range and amplitude than the late sodium current [11]. This raises the question whether the shifted activation and inactivation curves, observed in so many sodium channel mutations, could induce an additional heart-arrhythmia-promoting effect besides increasing the late sodium current.

A number of theoretical studies have related specific sodium channel mutations to the cardiac action potential shape by including all mutation-induced parameter changes simultaneously [5,12]. For any parameter besides the late sodium current, however, a systematic test of the impact on the cardiac action potential shape is currently missing. Here, we aim to fill this gap by analyzing the explicit consequences of changing sodium channel activation and inactivation curves and time-constants one by one. Using the used the reduced ten-Tusscher-model [13], we perform bifurcation studies for single epicardium ventricle cells with a focus on changes in the resting membrane potential and the action potential duration. Both parameters allow predictions about the impact on the signal transmission and heart arrhythmia induction [1,14,15] without requiring more complex simulations of signal propagation in space, which would depend on many additional and oftentimes not well-defined parameters.

Even though many sodium channel mutations that are associated with cardiac syndromes are known to affect the speed of the inactivation [4,5], our modeling results demonstrate that the cardiac action potential shape is quite robust to changes in the time-constants. In contrast, shifts of the activation curve to lower voltages and of the inactivation curve to higher voltages (which also can be observed in various sodium channel mutations) can induce fundamental changes in the voltage dynamics by inducing oscillations and bistability. As both, oscillations and bistability in the membrane potential, are known risk-factors for heart-arrhythmias [16,17], our study adds importance to other parameters than the late sodium current as possible risk-factors in certain cardiac syndromes.

## Methods

Cardiac action potential modeling was based on the reduced (9-variable) ten-Tusscher-model published by ten Tusscher and Panfilov in 2006 [13,18,19]. All simulations were performed for models of single epicardial ventricle cells with the original model equations and parameters.

We included four extra parameters (shift_ac,_ shift_inac,_ c_ac,_ c_inac_) in the differential equations for the sodium channel gates that define the kinetics of the sodium current

$$I_{Na}(V,m,h,j)=g_{Na}m^{3}hj(V-E_{Na}).$$

The new gating equations read:

$$\frac{dm(V)}{dt}=\frac{m_{\infty }(V+{shift}_{ac})-m}{\tau_{m}(V)∙c_{ac}},$$

$$\frac{dh(V)}{dt}=\frac{h_{\infty }(V+{shift}_{inac})-h}{\tau_{h}(V)∙c_{inac}},$$

$$\frac{dj(V)}{dt}=\frac{j_{\infty }(V+{shift}_{inac})-j}{\tau_{j}(V)∙c_{inac}}.$$

The parameters shift_ac_ and shift_inac_ control a shift in the voltage dependence of the sodium channel activation- and inactivation curves, respectively, while c_ac_ and c_inac_ are used to scale the sodium channel activation and inactivation time-constants.

Moreover, we chose a continuous description of the alpha and beta function for the h and j variables, thus replacing the differentiation in the two cases V>-40mV and V<-40mV by a logistic function *u(V)* that is continuous in the voltage:

$$u(V)=\frac{1}{1+e^{-5∙(V+40)}},$$

$$\alpha_{h}(V)=(1-u)∙0.057∙e^{-\frac{\left(V+80\right)}{6.8}},$$

$$\beta_{h}(V)=u∙\frac{0.77}{0.13∙(1+e^{-\frac{\left(V+10.66\right)}{11.1}})}+(1-u)∙(2.7∙e^{0.079∙V}+310000∙e^{0.3485∙V}),$$

$$\alpha_{j}(V)=u+(1-u)∙\frac{\left(\left(-25428∙e^{0.2444∙V})-(0.000006948∙e^{-0.04391∙V}\right))∙(V+37.78\right)}{1+e^{0.311∙(V+79.23)}},$$

$$\beta_{j}(V)=u∙\frac{0.6∙e^{0.057∙V}}{1+e^{-0.1∙(V+32)}}+(1-u)∙\frac{0.02424∙e^{-0.01052∙V}}{1+e^{-0.1378∙(V+40.14)}}.$$

The other model equations and parameters are unchanged from the original model paper [13]:

$$I_{ion}(V,m,h,j,s,x_{r1},xs,f,f_{2})=I_{Na}(V,m,h,j)+I_{K1}(V)+I_{to}(V,s)+I_{Kr}(V,x_{r1})+I_{Ks}(V,xs)+I_{Ca}(V,f,f_{1})+I_{NaCa}(V)+I_{NaK}(V)+I_{pCa}+I_{pK}(V)+I_{bCa}(V)+I_{bNa}(V),$$

$$\frac{dV}{dt}=\frac{I_{inj}(t)-I_{ion}}{C_{m}}.$$

A full list of all model equations and parameters can be found in the S1 Appendix.

For the injection current, we used repetitive rectangular pulses described by Heaviside step functions as an analytical, continuous approximation:

$$I_{inj}(t)=\frac{A}{\pi }\left[arctan(\frac{time(t)-t_{0}}{0.01})-arctan(\frac{time(t)-(t_{0}+l)}{0.01})\right]\;with\;time(t)=modulo(t,P).$$

The rectangular pulses with fixed amplitude (A), pulse length (l), and period (P) start after a time delay (t_0_). The modulo operation (which gives the remainder of the division of t and the period P) in the time description is important for the repetition of the signal. If not written otherwise, we used an amplitude of 60 pA/pF and a pulse length of l = 2 ms.

All simulations were performed with Python 2.7.13, using the integration solver odeint from Scipy 0.17.0.

For bifurcation analysis, we used the continuation and bifurcation software AUTO-07p.

The solutions of the AUTO-07p software are categorized in un-/stable nodes, foci, and limit cycles. Stable nodes represent a stable steady-state solution; the voltage converges to a stable resting membrane potential. Stable limit cycles represent a stable oscillatory solution of the membrane potential. Stable foci represent damped oscillations of the system which settle onto a stable steady-state (i.e. a stable resting potential). Unstable solutions are plotted for completeness (in the bifurcation diagram in Fig 3C) but cannot be seen in the corresponding membrane voltage traces, as any perturbation of the system always attracts the voltage dynamics away from the unstable solutions towards the stable solutions. In general, both, stability and the existence of fixpoints, can be read off the eigenvalues of the Jacobian matrix evaluated at the solution. Steady-state solutions are stable for a negative real part and unstable for a positive real part of the associated eigenvalues. Saddles and nodes correspond to fixpoints with no imaginary part. In contrast, non-vanishing imaginary parts indicate a stable or unstable focus. Limit cycle oscillations, starting with infinitesimal amplitude, can be found near Hopf bifurcations and are continued from there. More information about bifurcation and stability analysis can, for example, be found in the following textbooks [20,21].

## Results

### The cardiac action potential shape is robust to changes in sodium channel timeconstants

Our first goal was to study how the time scale of the sodium channel activation and inactivation can affect the cardiac action potential shape. Both, the activation and the inactivation time-constants, were multiplied by a factor of 0.01 to 100, rendering the channel gating 100 times slower or faster, respectively.

**Fig 1:**
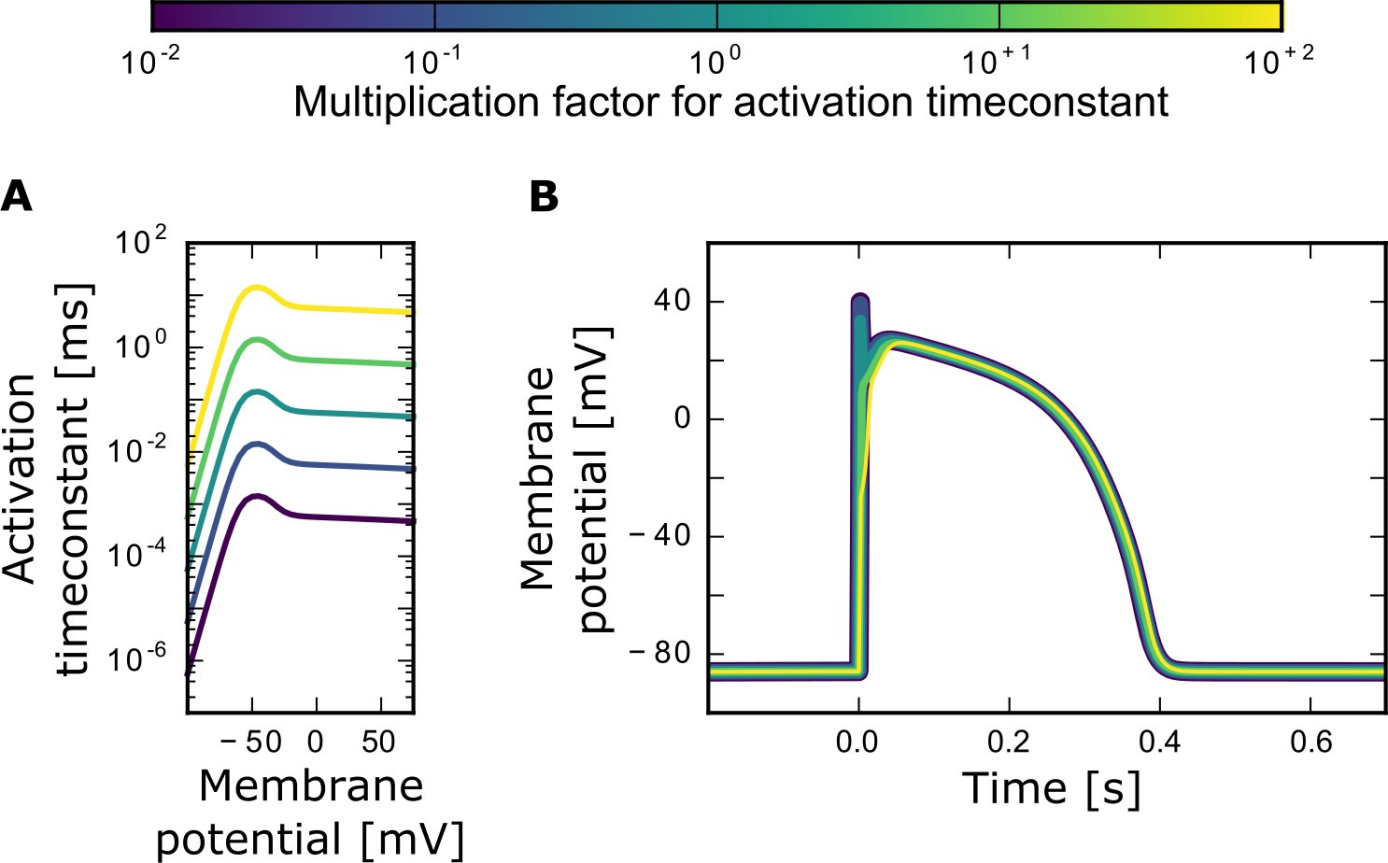
Changing the sodium channel activation time-constant τ_m_. (A) Different multiplication factors (from 10^−2^ to 10^+2^) result in different activation time-constants. (B) Different activation time-constants lead to different shapes of the cardiac action potential. The colors match the corresponding activation curve in A. The plot shows one action potential triggered by a 2 ms stimulation pulse of 60 pA/pF amplitude. The system was given 1 s to settle onto the resting potential before the pulse started at timepoint zero.

**Fig 2:**
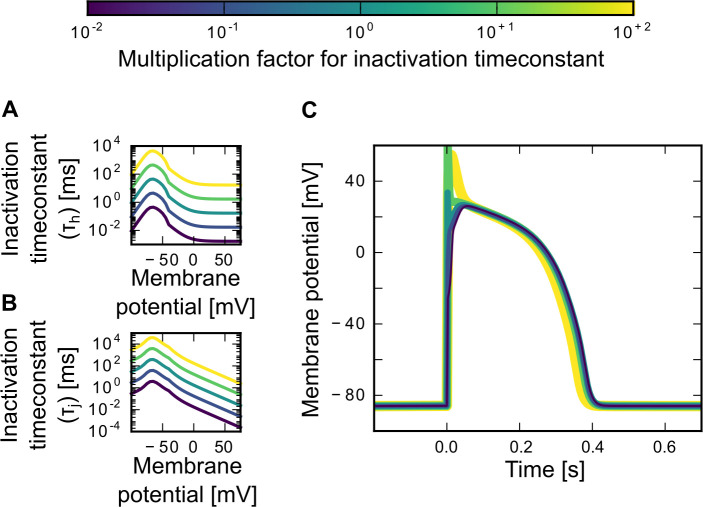
Changing the sodium channel activation time-*constant* τ_h_ *and* τ_j_. (A,B) Different multiplication factors (from 10^−2^ to 10^+2^) result in different time-constants for the sodium inactivation gates h (A) and j (B). (C) The different inactivation time-constants give rise to different shapes of the cardiac action potential. The colors match the corresponding inactivation curves in A and B. The plot shows one action potential triggered by a 2 ms stimulation pulse of 60 pA/pF amplitude. The system was given 1 s to settle onto the resting potential before the pulse started at timepoint zero.

Even though the voltage dependence of the activation time-constant τ_m_ (Fig 1A) and the inactivation time-constants τ_h_ (Fig 2A) and τ_j_ (Fig 2B) changed drastically, the overall action potential width and plateau height, as well as the resting potential, were similar across the broad range of different time-constants explored (Figs 1B and 2C). The main effect of the sodium time-constants on the action potential shape were related to the potential’s first peak. If the activation speed was high (i.e., the activation time-constant was small), so was the amplitude of the first peak. In contrast, a slow activation with large activation time-constant reduced the peak height and could even result in a complete loss of the first voltage peak (Fig 1B). For the inactivation time-constants the opposite was observed: small inactivation time-constants (fast inactivation) resulted in a loss of the first peak. An increase in the inactivation time-constants and, therefore, slow-down of the inactivation process, resulted in a higher peak amplitude of the first peak and also in its broadening (Fig 2C). Our results also showed that increasing the inactivation time-constant by a factor of 100 can lead to a small reduction in the overall action potential width.

### Shifts in the sodium channel activation curve can lead to oscillations in the membrane voltage

Another parameter which is often changed in mutated sodium channels associated with cardiac syndromes is the voltage dependence of the activation curve [4,5]. Often, mutations result in a shift of the voltage activation. To investigate the effects of such a modification, we systematically moved the activation curve by values in the range between -50 mV to +50 mV and analyzed changes in action potential shape and resting membrane potential. A shift to more positive voltages means the channel opens “later” in the course of the action potential, as higher depolarization levels are required. In contrast, a shift to more negative voltages results in an “earlier” opening of the sodium channel. The tested midpoint voltages ranged from -87.5 mV to 12.5 mV. For reference: in the artificial Na_V_1.5 sodium channel mutants described in Hsu et al. 2017 [6] the midpoint activation voltages range from −125.1 mV to -4.9 mV.

**Fig 3:**
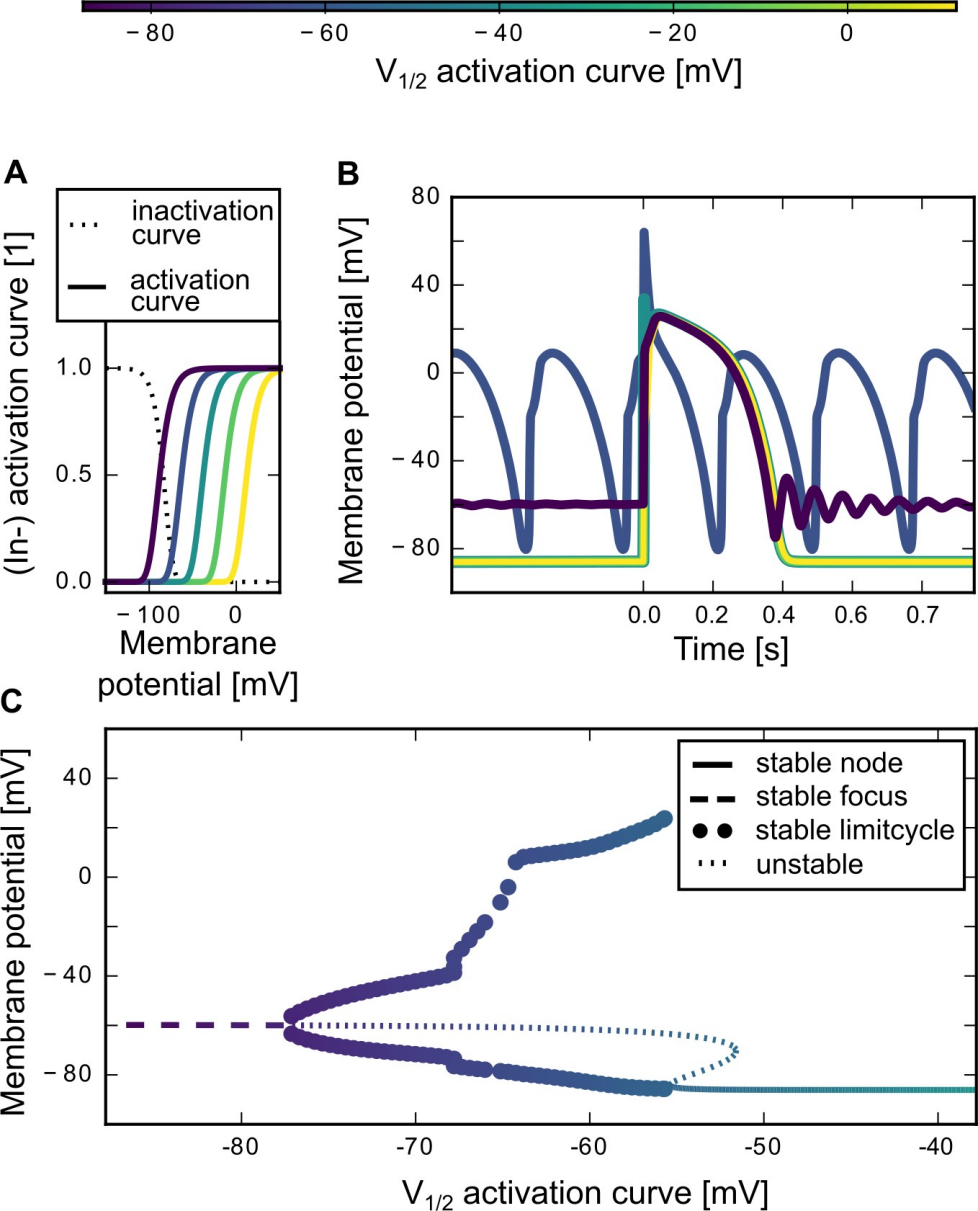
Shifting the sodium channel activation curve. (A) We shifted the midpoint voltage (V_1/2_) of the sodium channel activation curve from its original value at -37.8 mV in both directions: +50 mV to more positive and -50 mV to more negative voltages. The sodium channel inactivation curve remained unchanged. The activation and inactivation curves were based on the steady state values of the sodium activation and inactivation gate variables m, h and j (activation curve = m_∞_^3^, inactivation curve = h_∞_j_∞_). (B) Every shift of the sodium channel activation curve changed the cardiac action potential shape. The colors match the corresponding activation curve in A. The plot shows a part of a simulation with a 2 ms stimulation pulse of 60 pA/pF amplitude. The system was given 1 s to settle onto the resting potential before the pulse started at timepoint zero. (C) Bifurcation diagram which shows the resting potential of the system for negative shifts (-50 mV to 0 mV) in the midpoint voltage of the sodium activation curve. The linecolor matches the corresponding midpoint of the activation curve (x-axis), similar to the colorbar shown in the Fig and the color code of A and B.

Shifting the activation curve to more positive voltages (Fig 3A) had minor effects on the action potential shape. The first voltage peak vanished but action potential width, plateau height, and resting potential were unaffected (Fig 3B). Shifting the activation curve to more negative voltages, however, resulted in a qualitative switch in the voltage dynamics that included high-amplitude oscillations of the membrane potential that were not observable in the original (unshifted) model (Fig 3B). The occurrence of these oscillations indicates a qualitative change in the underlying dynamics. Mathematically, this change can be characterized in terms of so-called bifurcations of the dynamical system. The latter mark points of qualitative change in a system’s behaviour. Analysis of the underlying bifurcation structure allows one to better understand the dynamics and the observed properties (like oscillations). We therefore used the technique of numerical continuation to derive a diagram of the bifurcations as a function of the parameter that induced the change, i.e. the midpoint voltage of the activation curve. The diagram (Fig 3C) shows that a decrease of the activation curve’s midpoint voltage below ~-55 mV leads to the loss of the previously stable resting potential and the creation of a stable limit cycle, i.e. a self-sustained membrane-potential oscillation. The underlying bifurcation is a so-called saddle-node bifurcation. Therefore, in Fig 3B, we see a stable oscillation for the activation curve that is shifted by -25 mV (i.e., to the left, V_1/2_ = -62.5 mV). The shape of the oscillations differs from the normal shape of a pulse-induced action potential; the oscillations in the membrane potential continue even in the absence of stimulation. When the activation curve is shifted even further to the left, a collapse of the limit cycle can be observed at ~-77 mV (Fig 3C). Mathematically, this transition is a supercritical Hopf bifurcation. This means that the self-sustained membrane oscillations vanish and action potentials can again be elicited by pulse stimulation. Such action potentials lack the first voltage peak and the width and plateau height are only mildly changed. Also, at this point a stable focus appears, i.e. a resting membrane potential that is characterized by small, transient oscillations if mildly perturbed. The action potential is followed by damped membrane potential oscillations when it settles onto the focus (Fig 3B, V_1/2_ = -87.5 mV). The bifurcation diagram (Fig 3C) shows that the stable focus after the limit cycle collapse is at higher membrane voltages compared to the stable node before the limit cycle creation. Therefore, the resting membrane potential of the corresponding action potential (Fig 3B) is elevated. Moreover, the new resting membrane potential is a stable focus (Fig 3C), which means that the repolarization of the action potential results in a damped oscillation (Fig 3B).

### Shifts in the sodium channel inactivation curve can lead to bistability in the resting potential

**Fig 4:**
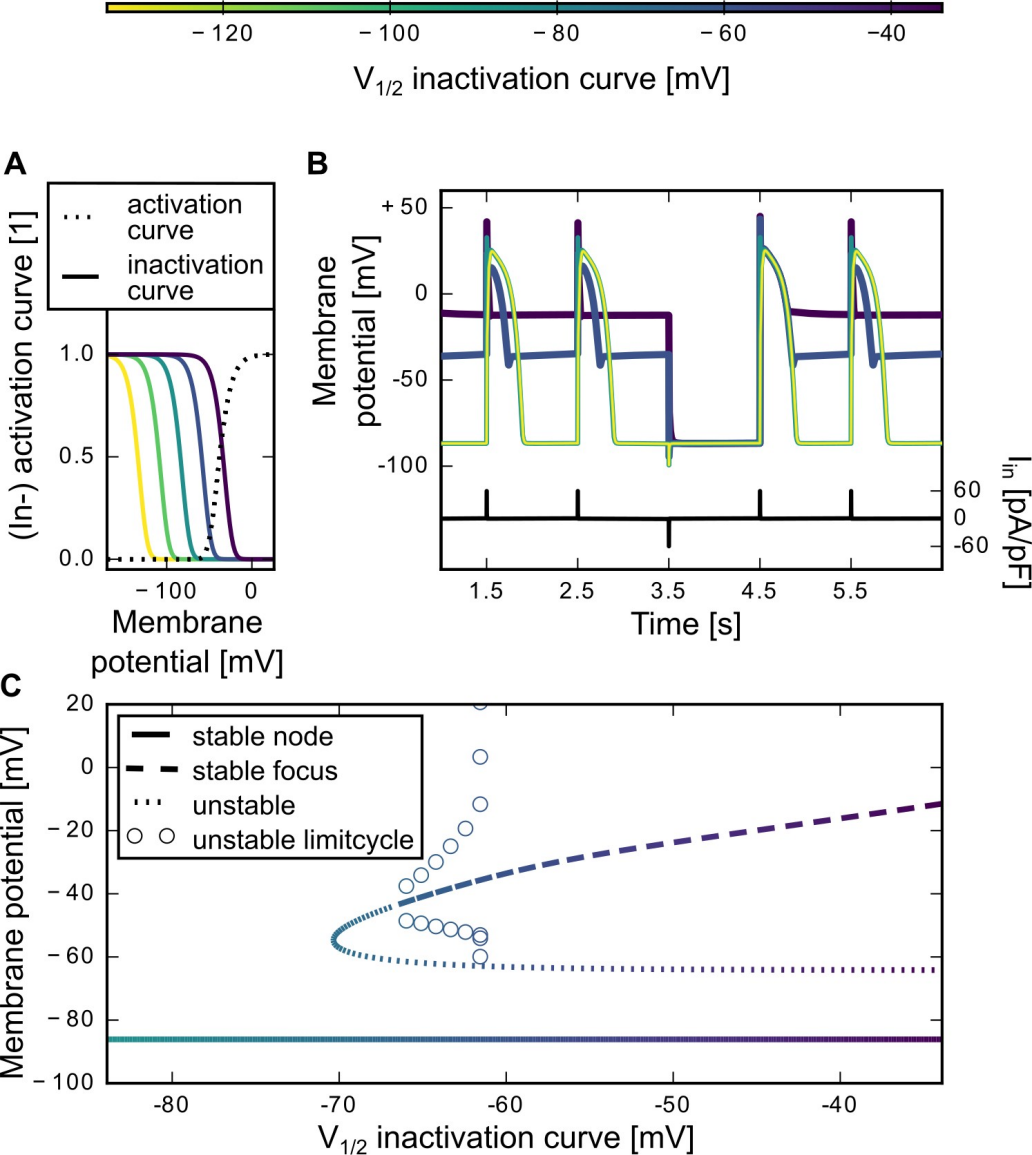
Shifting the sodium channel inactivation curve. (A) We shifted the midpoint voltage (V_1/2_) of the sodium channel inactivation curve from its original value at -83.9 mV in both directions: by +50 mV to more positive and -50mV to more negative voltages. The sodium channel activation curve remained unchanged. The activation and inactivation curves were based on the steady state values of the sodium activation and inactivation gate variables m, h and j (activation curve = m_∞_^3^, inactivation curve = h_∞_j_∞_). (B) Every shift of the sodium channel inactivation curve changed the cardiac action potential shape. The colors match the corresponding inactivation curve in A. The plot shows a part of a simulation with a stimulation pulse once every second (pulse length l = 2 ms, period P = 1 s, time delay t_0_ = 0.5 s). Pulse amplitude was always 60pA/pF (A = +60 pA/pF), just at t = 3.5 s the strength was reverted in sign (A(3.5 s) = -60 pA/pF). The current trace is shown in black. (C) Bifurcation diagram depicting the resting potential of the system for positive shifts (0 mV to 50 mV) in the midpoint voltage of the sodium inactivation curve. The linestyles code for the characteristics of the eigenvalues (see Methods). The linecolor matches the corresponding midpoint voltage of the inactivation curve (x-axis), also see colorbar.

Mutated sodium channels associated with cardiac syndromes tend to have shifted inactivation curves [4,5]. Therefore, we systematically moved the sodium inactivation curve by shifting the steady-state values of both inactivation gates, h and j, by the same amount (Fig 4A). Specifically, we covered a range of shifts from -50 mV (i.e., towards more negative values) to +50 mV (i.e. towards more positive voltages), resulting in midpoint inactivation voltages from -133.9 mV to -33.9 mV. We tested to what extent action potential shape and resting membrane potential were affected. For orientation: in the artificial Na_V_1.5 sodium channel mutants from Hsu et al. 2017 [6], the midpoint inactivation voltages ranged from −113.6 mV to -42.5 mV.

We next stimulated the model with a sequence of current pulses. For models with a negative shift of the inactivation curve, the inactivation started “earlier” in the course of an action potential. We observed only minor effects on the action potential shape (Fig 4B). The initial, fast peak decreased with negative shifts, but action potential width, plateau height, and resting potential remained mainly unaffected. In contrast, a positive shift of the sodium inactivation curve, corresponding to a “later” start of the inactivation in the action potential course, led to higher resting potentials (Fig 4B). A shift by +25mV (V_1/2_ = -58.9 mV) still resulted in a characteristic action potential shape with a first voltage peak and the plateau. However, the action potential width and the plateau height were reduced; the resting potential that followed a pulse in the stimulation was much higher (V_rest_ = ~-45 mV) compared to the corresponding resting potential observed in the original model (with unshifted inactivation), which was ~-86mV. Shifting the inactivation curve even further in the positive direction (V_1/2_ = -33.9 mV, Fig 4B), resulted in a progressive increase of the resting potential and a loss of the characteristic action potential shape. The corresponding bifurcation diagram (Fig 4C) shows that the stable node representing the resting potential in the unshifted, original model remained stable for positive shifts of the inactivation curve. However, if the midpoint voltage of the inactivation curve exceeded -66.9 mV; a second resting potential at higher voltages appeared. Because now two stable resting voltages co-existed in the same system, the latter was bistable. The upper stable resting potential emerged from a subcritical Hopf-bifurcation. The depolarization level of this second resting membrane potential gradually increased when shifting the sodium channel inactivation curve to the right (Fig 4C). Therefore, the resting membrane potential of the V_1/2_ = -33.9 mV example action potential trace in Fig 4B had a higher resting membrane potential than the V_1/2_ = -58.9 mV voltage trace. After action potentials triggered by positive current pulses, the system always repolarized to the elevated membrane potential (as it was the first stable equilibrium the system reached) and remained there until the next stimulation pulse. The system, however, is bistable: The original lower stable node (i.e. the lower resting potential) coexists. It could be accessed when reversing the sign of the injection current. In Fig 4B, this behaviour is illustrated by the third stimulation pulse, which in contrast to the other ones was negative. In response to this negative pulse, the membrane voltage jumped back to the (original) lower resting membrane potential (and not the higher one, as it did for the positive pulses).

### Shifting both the activation and inactivation curves simultaneously

So far, we only changed activation or inactivation at a time. In physiology, however, sodium channel mutations can exhibit modifications in both, the activation and inactivation curves and their interaction is unclear. As we have seen above, most interesting are qualitative changes in voltage dynamics, like the occurrence of membrane potential oscillations or membrane potential bistability, which we here term critical (as they both bear relevance of cardiac arrhythmicity). In particular, it could be that the effects of shifts of activation and inactivation curves cancel out, when both curves are effected and no critical behaviour of the membrane potential is observed. Although this is a valid possibility, we here present an example illustrating that critical behavior is still possible.

Combining changes in both curves, we may expect nonlinear interactions between the effects of both curves. If both curves were shifted to more negative voltages, the activation curve—when viewed in isolation—could be in the critical oscillatory regime, while the inactivation curve would be further away from its critical bistable regime. In contrast, if both, the activation and inactivation curves were shifted to more positive voltages, the activation curve would be less critical (in its ability to exhibit resting-potential oscillations). The inactivation curve, however, would be more prone to induce bistability.

**Fig 5:**
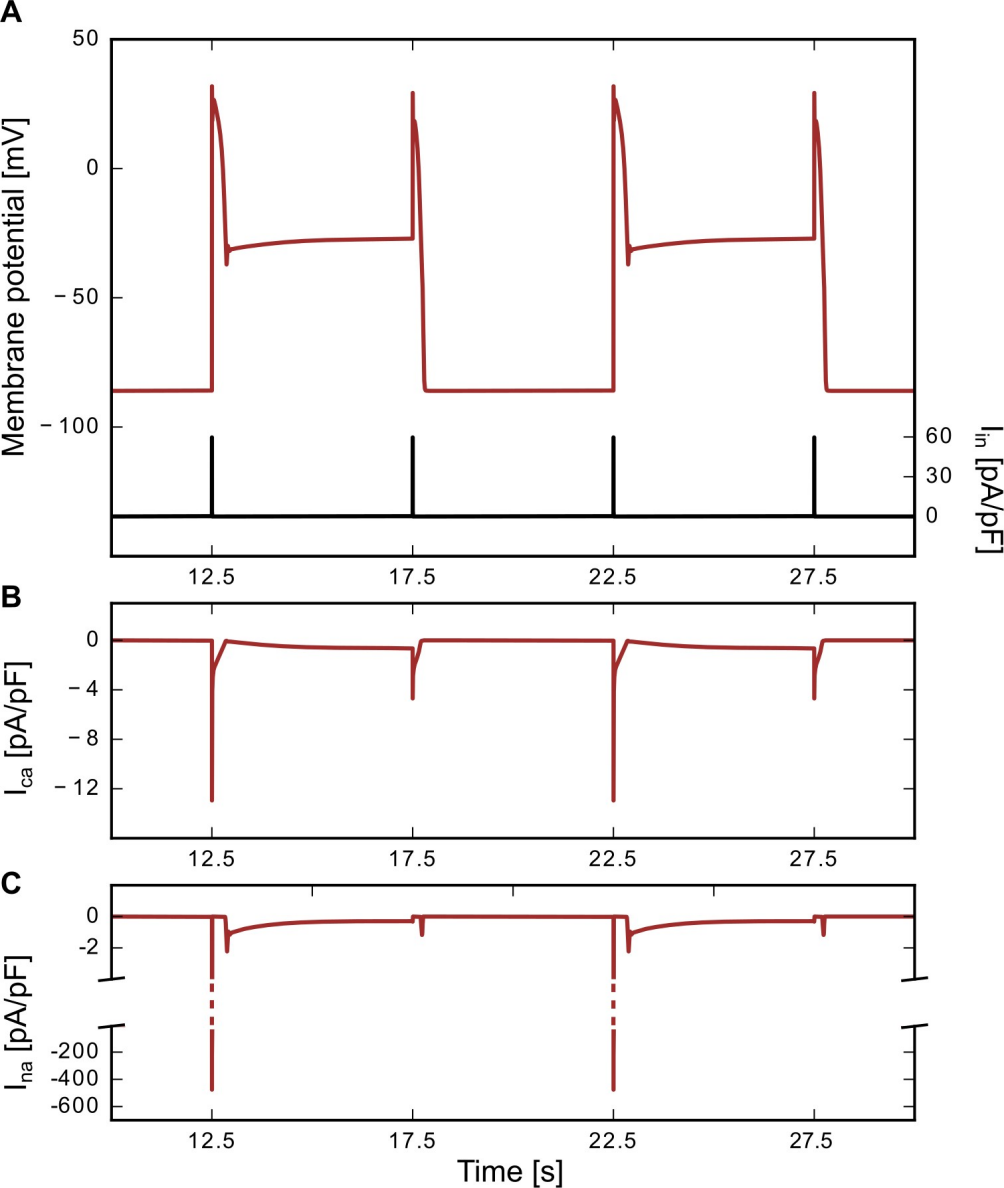
Example trace for N1659A mutation. (A) Action potential trace for the N1659A sodium channel mutants activation and inactivation curve [6]. The midpoint voltage of the activation curve was set to V_1/2_ = -23.7 mV and the midpoint-voltage of the inactivation curve, was set to V_1/2_ = -52.6 mV. The plot shows a part of a simulation with a stimulation pulse every 5 s (pulse amplitude A = 60 pA/pF, pulse length l = 2 ms, period P = 5 s, time delay t_0_ = 2.5 s). The current trace is included (black line). (B) Calcium and (C) sodium current traces corresponding to the action potential simulations in A.

To choose a physiological example, we focussed on the artificial sodium channel mutant N1659A which is listed in the study by Hsu et al. [6]. The simulation of this N1659A sodium channel is interesting because its midpoint for the inactivation curve is shifted to positive voltage values and (with -52.6 ±1.5 mV) lies in the bistable regime predicted by our simulations. Its activation curve, however, is also positively shifted with a midpoint voltage of V_1/2_ = -23.7 ±2.4 mV and, therefore, even further away from the model-predicted oscillatory regime than the wildtype Na_V_1.5 channel. Incorporating these characteristics into the model, we indeed observed a critical, history-dependent switching behavior in the voltage-trace (Fig 5A). The membrane voltage changed from one of two stable resting potentials with each new stimulation pulse. Interestingly, the sign of the stimulation pulse did not even have to change to induce a switch to the other resting potential (compare to Fig 4B, where pulses of opposite signs were needed for the switch). Each switch in resting potential was accompanied by a change in the shape of the next action potential shape. Action potentials elicited from the lower resting potential exhibited an increased width and amplitude compared to the action potentials triggered from the higher resting potential. Both cases do not only differ in the action potential shape but also in the underlying ionic currents. Fig 5B and 5C depict the associated sodium and calcium currents. When the resting potential was elevated, the sodium current was reduced and the action potential was mainly calcium-driven, while in the lower resting potential case, the sodium potential was the leading current for the initial depolarization. Thus critical dynamics, as we observed them for isolated changes in either activation or inactivation curves can also—in modified form—be observed when both types of curves are affected simultaneously. The changes in voltage dependence of both curves and their different combinations are thus likely to endow different channel mutants with interesting critical behaviours.

## Discussion

In this study, we investigated the effect of sodium channel parameters on the cardiac action potential shape. We used the reduced ten-Tusscher-model for single ventricular epicardium cells [13] and systematically varied the sodium channel activation and inactivation time-constants as well as midpoint voltages. These parameters are well known to be altered in sodium channel mutations that have been associated with diverse cardiac syndromes. Nevertheless, the potential of these parameters to induce heart-arrhythmias has not been acknowledged in previous studies, presumably because of the medical focus on the persistent (late) sodium current as the most crucial parameter in cardiac syndromes.

### Minor effects of sodium channel time-constants

Our modelling results suggest that the cardiac action potential shape is quite robust to changes in the sodium channel time-constants. In the cardiac action potential, the sodium current is important for the initiation of the cardiac action potential. It is needed for the fast initial rise of the cardiac action potential, while the calcium and various potassium currents are more relevant for the cardiac action potential plateau and repolarization. This fact provides an intuitive explanation for why changes in the sodium channel time-constants of five orders of magnitude had only minor effects on the overall action potential shape. While action potential rise time and the height of the first sodium current-induced peak in the membrane potential were affected, the action potential plateau and width remained robust. For completeness, however, we note that the observed changes in initial voltage height and rise time may play a role in the signal conduction from cell to cell–a question not addressed in this study.

### Shifts in the activation curve can lead to oscillatory behavior

Various sodium channel mutations, associated with different cardiac syndromes, are characterized by shifts in the voltage dependence of the activation curve, which may contribute to arrhythmic dynamics. In our model, shifts to more positive voltages had qualitatively similar effects to increases of the activation time-constant or decreases of the inactivation time-constant: the initial peak of the cardiac action potential was lost, but plateau, action potential width, and resting potential were mainly unaffected. A strong negative shift of the sodium channel activation curve (V_1/2_ < -77 mV) also resulted in a lack of the first sodium peak while the action potential width and plateau was unchanged. A clear difference between positive and negative shifts in the voltage dependence of activation was the elevated resting membrane potential in the latter case. Moreover, for strongly negative shifts the return to the resting potential after an action potential was accompanied by unstable oscillations with decaying amplitude.

In our simulation results, however, the most fundamental change in the cardiac action potential shape occurred with negative activation-curve shifts of intermediate size (-77 mV < V_1/2_ < -55 mV). In this parameter region we observed an oscillatory behavior of the membrane voltage. From a dynamical system’s perspective, the stable steady-state of the resting membrane potential lost stability when the sodium channel was activated at lower voltages. A stable limit cycle appeared, which resulted in periodic depolarizations in the absence of input. Spontaneous (stimulus-independent) depolarization in the absence of stimulation and under physiological conditions has so far only been reported in the node-tissues. Abnormal automaticity [17,22], however, can for example happen in the ventriculum when the resting potential and the ionic environment are altered. Our model analysis suggest that also negative shifts of the sodium channel activation curve can trigger abnormal automaticity and therefore constitute a risk factor for heart-arrhythmias.

### Shifts in the inactivation curve can lead to bistability

Also shifts in the voltage-dependence of the sodium channel inactivation have been associated with cardiac pathologies, such as Long-Q-T or Brugada syndromes [4,5]. Our analysis demonstrates that positive shifts in the inactivation curve (i.e. to higher voltages), result in a bistability in the resting potential. If the voltage of midpoint activation is around -65 mV or higher, there were two stable resting potentials instead of only one. The additional stable resting potential has a more depolarized voltage value (-50 mV to -10 mV) than the co-existing “original” resting potential (-89 mV). The new stable resting potential is always reached after a cardiac action potential, which terminates at this voltage. As expected for the system with two stable attractors, injection of hyperpolarizing current pulses induces a transition to the lower resting potential. We note that the voltage value of the additional stable resting potential depended on the on the midpoint voltage of the inactivation curve. Such bistability in the resting potential has been previously experimentally observed in cardiac electrophysiology. In 1977, Gadsby and Cranefield created bistable resting potentials in Purkinje fiber cells of dogs by changing the extracellular potassium concentration [23]. Similar to our simulation results, they induced jumps between a low resting potential around -91 mV and a higher resting potential around -49 mV by injection current pulses of opposite signs.

Bistability in the resting potential can be a risk factor for heart arrhythmias because it can lead to bistable wave propagation, also called “biexcitability” [24]. The lower resting potential gives rise to action potentials which are initiated by both sodium and calcium currents, while the upper resting potential give rise to slower, calcium-driven action-potentials. A previous study from Chang et al. established a bistability in the resting potential by simultaneous modification of many parameters in their rabbit ventricular model, pushing the system into a regime where early afterdepolarization occur [16]. Our model shows that shifts in the voltage dependence of sodium channel inactivation suffice to induce a bistability in the resting membrane potential.

### Factors that can shift the (in-)activation curve

Our simulations predict drastic changes in the voltage dynamics if the midpoint voltage of the sodium channel activation curve crosses a lower boundary (< -55 mV in our model) and/or the midpoint voltage of the inactivation is significantly increased (> -65 mV in our model). Hsu et al. [6] reviewed the contribution of the different sodium channel domains to the sodium channel inactivation process. Interestingly, from the 29 sodium channel variants listed there, 21 fulfill at least one of the two voltage boundary criteria. This shows that experimentally characterized sodium channel mutations can easily reach the critical regime of activation- or inactivation voltage dependence predicted by our model.

In view of the relevance of shifts in voltage dependence, the question arises whether there are other factors besides channel mutations that affect these parameters and therefore modulate the risk for heart arrhythmias. Indeed, anti-arrhythmic drugs like lidocaine are known to produce a negative (i.e., leftwards) shift of the sodium channel inactivation curve [25]. Our results suggest an explanation for this rhythm-stabilizing effect, as it opposes positive (i.e., rightwards) shifts of the sodium channel inactivation curve, the latter of which we predict to increase the risk for bistability and, therefore, the risk for arrhythmic heart beats. Also general physiological parameters, like temperature, can shift the voltage-dependence of sodium channel activation and inactivation. If the temperature sensitivity of opening and closing rates of an ion channel gate are different, temperature increases do not only speed up gating but also result in a shift in the voltage-dependence of the activation (or inactivation). The experimental study of Abdelsayed et al. [5] shows that increasing temperature can produce a left shift (towards lower voltages) in both the sodium channel activation and inactivation curves. They also report a differential temperature dependence of different sodium channel mutants. Our model analysis suggests an increased risk for self-sustained oscillations for left-shifted activation curves. This result contributes a mechanistic explanation why also changes in temperature can trigger heart-arrhythmias in cardiac pathologies like the Brugada-syndrome.

### Summary

Taken together, our study demonstrates the relevance of shifts in the voltage-dependence of sodium channel activation and inactivation kinetics for the properties of the cardiac action potential–suggesting these properties as relevant inducers next to the more widely discussed effect of the persistent sodium current. The consequences of changes in the activation and inactivation voltage dependence suffice to explain an increased risk for heart arrhythmias because of their ability to induce oscillatory voltage dynamics as well as membrane-potential bistability and thus provide a novel mechanistic explanation that should be taken into account in medical research.

## Supporting information

S1 Appendix (DOCX)
