# Supplementary material for: Modifications of sodium channel voltage dependence induce arrhythmia-favouring dynamics of cardiac action potentials
Source: PLoS One. 2020 Aug 4;15(8):e0236949. doi: 10.1371/journal.pone.0236949 (PMC7402508; doi:10.1371/journal.pone.0236949)
Supplement: S1 Appendix — (DOCX) [file pone.0236949.s001.docx]

# **Appendix**

Based on the reduced ten-Tusscher model (13), the following model equations and parameters were used:

$m_{\infty}=\frac{1}{\left( 1+e^{\frac{-56.86-V}{9.03}} \right)^{2}}$,

$\tau_{m}\left( V \right)=\frac{1}{1+e^{\frac{-60-V}{5}}}\cdot\left( \frac{0.1}{1+e^{\frac{V+35}{5}}}+\frac{0.1}{1+e^{\frac{V-50}{200}}} \right)$,

$u(V)= \frac{1}{1+e^{-5\cdot(V+40)}}$,

$\alpha_{h}(V)=u\cdot0+\left( 1-u \right)\cdot0.057\cdot e^{-\frac{(V+80)}{6.8}}$,

$\beta_{h}(V)=u\cdot\frac{0.77}{0.13\cdot\left( 1+e^{-\frac{\left( V+10.66 \right)}{11.1}} \right)}+\left( 1-u \right)\cdot\left( 2.7\cdot e^{0.079\cdot V}+310000\cdot e^{0.3485\cdot V} \right)$,

$h_{\infty}\left( V \right)=\frac{1}{\left( 1+e^{\frac{V+71.55}{7.43}} \right)^{2}}$,

$\tau_{h}=\frac{1}{\alpha_{h}(V)+\beta_{h}(V)}$,

$\alpha_{j}(V)=u\cdot0+(1-u)\frac{\left( \left( -25428\cdot e^{0.2444\cdot V} \right)-\left( 0.000006948\cdot e^{-0.04391\cdot V} \right) \right)\cdot\left( V+37.78 \right)}{1+e^{0.311\cdot(V+79.23)}}$,

$\beta_{j}(V)=u\cdot\frac{0.6\cdot e^{0.057\cdot V}}{1+e^{-0.1\cdot\left( V+32 \right)}}+\left( 1-u \right)\cdot\frac{0.02424\cdot e^{-0.01052\cdot V}}{1+e^{-0.1378\cdot(V+40.14)}}$,

$j_{\infty}\left( V \right)=\frac{1}{\left( 1+e^{\frac{V+71.55}{7.43}} \right)^{2}}$,

$\tau_{j}=\frac{1}{\alpha_{j}(V)+\beta_{j}(V)}$,

$I_{Na}\left( V,m,h,j \right)=g_{Na}m^{3}hj\left( V-E_{Na} \right)$,

$d_{\infty}\left( V \right)=\frac{1}{1+e^{\frac{-8-V}{7.5}}}$,

$f_{\infty}\left( V \right)=\frac{1}{1+e^{\frac{V+20}{7}}}$,

$\alpha_{f}(V)=1102.5\cdot e^{-\left( \left( \frac{V+27}{15} \right)^{2} \right)}$,

$\beta_{f}\left( V \right)=\frac{200}{1+e^{\frac{13-V}{10}}}$,

$\gamma_{f}\left( V \right)=\frac{180}{1+e^{\frac{V+30}{10}}}+20$,

$\tau_{f}\left( V \right)=\alpha_{f}(V)+\beta_{f}(V)+\gamma_{f}(V)$,

${f_{2}}_{\infty}\left( V \right)=\frac{0.67}{1+e^{\frac{V+35}{7}}}+0.33$,

$\alpha_{f_{2}}\left( V \right)=600\cdot e^{-\frac{\left( V+27 \right)^{2}}{170}}$,

$\beta_{f_{2}}\left( V \right)=\frac{7.75}{1+e^{\frac{25-V}{10}}}$,

$\gamma_{f_{2}}\left( V \right)=\frac{16}{1+e^{\frac{V+30}{10}}}$,

$\tau_{f_{2}}\left( V \right)=\alpha_{f_{2}}(V)+\beta_{f_{2}}(V)+\gamma_{f_{2}}(V)$,

$I_{Ca}\left( V,f,f_{2} \right)=g_{Ca}\cdot d_{\infty}(V)\cdot f\cdot f_{2}\cdot\left( V-60 \right)$,

$r_{\infty}\left( V \right)=\frac{1}{1+e^{\frac{20-V}{6}}}$,

$s_{\infty}\left( V \right)=\frac{1}{1+e^{\frac{V+20}{5}}}$,

$\tau_{s}\left( V \right)=85\cdot e^{-\frac{\left( V+45 \right)^{2}}{320}}+\frac{5}{1+e^{\frac{V-20}{5}}}+3$,

$I_{to}\left( V,s \right)=g_{to}\cdot r_{\infty}\cdot s\cdot\left( V-E_{K} \right)$,

${xs}_{\infty}\left( V \right)=\frac{1}{1+e^{\frac{-5-V}{14}}}$,

$\alpha_{xs}\left( V \right)=\frac{1400}{\sqrt{1+e^{\frac{5-V}{6}}}}$,

$\beta_{xs}\left( V \right)=\frac{1}{1+e^{\frac{V-35}{15}}}$,

$\tau_{xs}\left( V \right)=\left( \alpha_{xs}(V)\cdot\beta_{xs}(V) \right)+80$,

$I_{ks}\left( V,xs \right)=g_{ks}\cdot{xs}^{2}\cdot\left( V-E_{ks} \right)$,

${x_{r1}}_{\infty}\left( V \right)=\frac{1}{1+e^{\frac{-26-V}{7}}}$,

$\alpha_{x_{r1}}\left( V \right)=\frac{450}{1+e^{\frac{-45-V}{10}}}$,

$\beta_{x_{r1}}\left( V \right)=\frac{6}{1+e^{\frac{V+30}{11.5}}}$,

$\tau_{x_{r1}}\left( V \right)=\alpha_{x_{r1}}(V)\cdot\beta_{x_{r1}}(V)$,

$x_{{r2}_{\infty}}\left( V \right)=\frac{1}{1+e^{\frac{V+88}{24}}}$,

$I_{kr}\left( V,x_{r1} \right)=g_{kr}\cdot x_{r1}\cdot x_{{r2}_{inf}}(V)\cdot\left( V-E_{K} \right)$,

$\alpha_{k1}\left( V \right)=\frac{0.1}{e^{0.06\cdot\left( V-E_{K}-200 \right)}}$,

$\beta_{k1}\left( V \right)=3\cdot e^{0.0002\cdot\left( V-E_{K}+100 \right)}+\frac{e^{0.1\cdot\left( V-E_{K}-10 \right)}}{1+e^{-0.5\cdot\left( V-E_{K} \right)}}$,

$x_{{k1}_{\infty}}\left( V \right)=\frac{\alpha_{k1}(V)}{{\alpha_{k1}\left( V \right)+\beta}_{k1}\left( V \right)}$,

$I_{k1}\left( V \right)=g_{k1}\cdot\sqrt{\frac{K_{o}}{5.4}}\cdot x_{{k1}_{inf}}(V)\cdot\left( V-E_{K} \right)$,

$a_{1}(V)=e^{\frac{\gamma\cdot V\cdot F}{R\cdot T}}\cdot{{Na}_{i}}^{3}\cdot{Ca}_{o}$,

$a_{2}\left( V \right)=e^{\frac{(\gamma-1)\cdot V\cdot F}{R\cdot T}}\cdot{{Na}_{o}}^{3}\cdot{Ca}_{i}\cdot\alpha$,

$b_{1}={{km}_{Na_{i}}}^{3}+{{Na}_{o}}^{3}$,

$b_{2}={km}_{Ca}+{Ca}_{o}$,

$b_{3}(V)=1+k_{sat}\cdot e^{\frac{(\gamma-1)\cdot V\cdot F}{R\cdot T}}$,

$I_{NaCa}\left( V \right)=k_{NaCa}\cdot\frac{a_{1}(V)-a_{2}(V)}{b_{1}\cdot b_{2}\cdot b_{3}(V)}$,

$I_{NaK}\left( V \right)=p_{NaK}\cdot\frac{K_{o}\cdot{Na}_{i}}{\left( K_{o}+{km}_{K} \right)\cdot({Na}_{i}+{km}_{Na})\cdot\left( 1+\left( 0.1245\cdot e^{\frac{-0.1\cdot V\cdot F}{R\cdot T}} \right)+\left( 0.0353{\cdot e}^{\frac{-V\cdot F}{R\cdot T}} \right) \right)}$,

$I_{pCa}=g_{pca}\cdot\left( \frac{{Ca}_{i}}{k_{pCa}+{Ca}_{i}} \right)$,

$I_{pK}(V)=g_{pK}\cdot\frac{V-E_{K}}{1+e^{\frac{25-V}{5.98}}}$,

$I_{bNA}\left( V \right)=g_{bNa}\cdot\left( V-E_{Na} \right)$,

$I_{bCa}\left( V \right)=g_{bCa}\cdot\left( V-E_{Ca} \right)$,

$I_{ion}\left( V,m,h,j,s,x_{r1},xs,f,f_{2} \right)=I_{Na}\left( V,m,h,j \right)+I_{K1}\left( V \right)+I_{to}\left( V,s \right)+I_{Kr}\left( V,x_{r1} \right)+I_{Ks}\left( V,xs \right)+I_{Ca}\left( V,f,f_{1} \right)+I_{NaCa}\left( V \right)+I_{NaK}\left( V \right)+I_{pCa}+I_{pK}(V)+I_{bCa}(V)+I_{bNa}(V)$,

$\frac{dV}{dt}=\frac{I_{inj}\left( t \right)-I_{ion}}{C_{m}}$,

$$\frac{dm(V)}{\mathrm{dt}}= \frac{m_{\infty}\left( V+\mathrm{shift}_{\mathrm{ac}} \right)-m}{\tau_{m}(V)\cdot c_{\mathrm{ac}}},$$

$\frac{dh(V)}{\mathrm{dt}}= \frac{h_{\infty}\left( V+\mathrm{shift}_{\mathrm{inac}} \right)-h}{\tau_{h}(V)\cdot c_{\mathrm{inac}}}$,

$\frac{dj(V)}{\mathrm{dt}}= \frac{j_{\infty}\left( V+\mathrm{shift}_{\mathrm{inac}} \right)-j}{\tau_{j}(V)\cdot c_{\mathrm{inac}}}$,

$$\frac{ds}{dt}=\frac{s_{\infty}\left( V \right)-s}{\tau_{s}},$$

$\frac{dx_{r1}}{dt}=\frac{{x_{r1}}_{\infty}\left( V \right)-x_{r1}}{\tau_{x_{r1}}}$,

$\frac{dxs}{dt}=\frac{{xs}_{\infty}\left( V \right)-xs}{\tau_{xs}}$,

$\frac{df}{dt}=\frac{f_{\infty}\left( V \right)-f}{\tau_{f}}$,

$\frac{df_{2}}{dt}=\frac{{f_{2}}_{\infty}\left( V \right)-f_{2}}{\tau_{f_{2}}}$,

$E_{K}=\frac{R\cdot T}{F}\cdot log\left( \frac{K_{o}}{K_{i}} \right)$,

$E_{Na}=\frac{R\cdot T}{F}\cdot log\left( \frac{{Na}_{o}}{{Na}_{i}} \right)$,

$E_{Ca}=\frac{R\cdot T}{F}\cdot log\left( \frac{{Ca}_{o}}{{Ca}_{i}} \right)$,

$E_{Ks}=\frac{R\cdot T}{F}\cdot log\left( \frac{K_{o}+\left( p_{KNa}\cdot{Na}_{o} \right)}{K_{i}+\left( p_{kNa}\cdot{Na}_{i} \right)} \right)$.

The parameters were:

| R | 8.3143 J/molK |
| --- | --- |
| F | 96.48533 |
| T | 310 K |
| C_m_ | 2 µF/cm^2^ |
| K_o_ | 5.4 mM |
| Na_o_ | 140 mM |
| Ca_o_ | 2 mM |
| K_i_ | 138.3 mM |
| Na_i_ | 7.67 mM |
| Ca_i_ | 0.00007 mM |
| g_Na_ | 14.838 nS/pF |
| g_K1_ | 5.405 nS/pF |
| g_to_ | 0.294 nS/pF |
| g_Kr_ | 0.101 nS/pF |
| g_Ks_ | 0.257 nS/pF |
| p_KNa_ | 0.03 |
| g_Ca_ | 0.2786 cm3/µFs |
| k_NaCa_ | 1e+3 pA/pF |
| γ | 0.35 |
| km_Ca_ | 1.38 mM |
| km_Nai_ | 87.5 mM |
| k_sat_ | 0.1 |
| α | 2.5 |
| p_NaK_ | 2.724 pA/pF |
| km_K_ | 1 mM |
| km_Na_ | 40 mM |
| g_pK_ | 0.0293 ns/pF |
| g_pCa_ | 0.1238 nS/pF |
| k_pCa_ | 0.0005 mM |
| g_bNa_ | 0.00029 nS/pF |
| g_bCa_ | 0.000592 nS/pF |
